# Protein kinase C activation upregulates human L-type amino acid transporter 2 function

**DOI:** 10.1186/s12576-021-00795-0

**Published:** 2021-03-31

**Authors:** Hanae Morio, Yoshie Reien, Yuri Hirayama, Hirofumi Hashimoto, Naohiko Anzai

**Affiliations:** 1 Department of Pharmacology, Graduate School of Medicine, Chiba University, 1-8-1 Inohana, Chuou-ku, Chiba, 260-8670 Japan; 2 Department of Pharmacology and Toxicology, School of Medicine, Dokkyo Medical University, 880 Kitakobayashi, Mibu-cho, Shimotsuga-gun, Tochigi, 321-0293 Japan

**Keywords:** L-type amino acid transporter 2, SLC7A8, Protein kinase C, Amino acids, Phosphorylation

## Abstract

L-type amino acid transporter 2 (LAT2) is a Na^+^-independent neutral amino acid transporter, whose function regulation system remains unclarified. Since protein kinase C (PKC) is known to regulate the functions of various transporters, we investigated whether human LAT2 (hLAT2) function is regulated by PKC. In mouse proximal tubule S2 cells, hLAT2 transport activity was upregulated by PKC activation. However, we found that the mRNA and protein expression of hLAT2 was not affected by PKC activation and that the upregulation was independent of the three potential PKC consensus sites in the hLAT2 amino acid sequence. Moreover, we found that PKC activation upregulated the *Vmax* value for hLAT2-mediated alanine transport, which was not accompanied by the induction of hLAT2 membrane insertion. In conclusion, we showed that hLAT2 function is upregulated by PKC activation, which is not related to either the de novo synthesis, the phosphorylation or the membrane insertion of hLAT2.

**Supplementary Information:**

The online version contains supplementary material available at 10.1186/s12576-021-00795-0.

## Background

L-type amino acid transporter 2 (LAT2) is a Na^+^-independent neutral amino acid transporter for which functional expression requires conformation of the heterodimer with 4F2 heavy chain antigen (4F2hc) [1, 2]. There is another LAT family member, LAT1, which also associates with 4F2hc to form a heterodimer [3]. However, characteristics of LAT2, including its substrate specificity and its expression profile, are different from those of LAT1. LAT2 displays broader substrate selectivity range than LAT1 and is responsible for the exchange of a wide range of amino acids including almost all neutral amino acids (except proline) [1, 2], which are important nutrients and signal factors for cell growth. Amino acid-mimic drug such as L-DOPA [4] and some hormones such as thyroid hormones [5] are also known as its substrates. While LAT1 is strongly expressed in the human brain, placenta and various kinds of tumors [3, 6, 7], LAT2 is known to be widely expressed in human tissues including the small intestine, kidney, placenta, skeletal muscle and brain [2, 6, 8]. Among these tissues, LAT2 is mainly expressed in the proximal tubule of the kidney and intestine, and it has been shown to be expressed at the basolateral membrane in epithelial cells [1, 8, 9]. Thus, LAT2 is recognized as an important factor relating to (re) absorption of its substances to blood. Previous studies showing dysfunction of LAT2 in several diseases further supports its importance in maintaining homeostasis [10, 11]. Together with the importance of LAT2 in normal cells, it has also been reported that LAT2 is related to some diseases. LAT2 has been shown to be over-expressed in the kidneys of spontaneously hypertensive rats compared to its expression in normotensive rats, together with enhanced uptake of L-DOPA [12]. Since L-DOPA is known to be converted to dopamine in proximal tubule cells and raise blood pressure, this report indicates the possibility that LAT2 can be the important factor that is related to the onset of hypertension. Despite the importance of LAT2 in both normal cells and abnormal cells, little is known about the regulatory mechanisms by which its expression and function are controlled.

As one of the candidates that may control LAT2 function, we focused on protein kinase C (PKC). PKC, which is one of the serine/threonine kinases, is involved in the mediation of responses to many factors and acts as a critical regulator of numerous cellular functions including cell cycle, migration, differentiation, morphogenesis, cell survival and apoptosis [13]. It is known that PKC can regulate the functions of various transporters [14], and it has also been reported that the function of LAT family is modulated by PKC [15–17]. However, the effect of PKC on LAT2 function has not been clarified yet. Therefore, the aim of the present study was to determine whether hLAT2 function is regulated by PKC.

## Materials and methods

### Cells and cell culture

Immortalized proximal tubule cell line established from transgenic mice harboring the temperature-sensitive SV40 large T-antigen gene (named S2 cells) [18], S2 cells stably expressing human LAT2 (S2-LAT2), and its mock cells (S2-Mock) [19] were used in the present study.

S2 cells, S2-Mock cells and S2-LAT2 cells were cultured in Dulbecco's Modified Eagle's Medium (DMEM)/Ham’s F-12 (Nacalai Tesque, Kyoto, Japan). Culture mediums were supplemented with 5% (v/v) heat-inactivated fetal bovine serum (FBS), 1% (v/v) insulin-transferrin-selenium supplement (Thermo Fisher Scientific, Waltham, MA), recombinant human epidermal growth factor (10 ng/mL) (Sigma-Aldrich, St. Louis, MO), and antibiotics. For stable cell lines, G418 (500 µg/mL) (Nacalai Tesque) was additionally supplemented for selection. All S2 cell lines were routinely grown at 33 ºC in a 5% CO_2_ atmosphere.

Caco-2 cells, human colorectal cancer cells, were obtained from DS Pharma Biomedical. Caco-2 cells were cultured in DMEM (High Glucose) (FUJIFILM Wako Pure Chemical Corp., Kyoto, Japan), supplemented with 10% (v/v) FBS and antibiotics. Caco-2 cells were routinely grown at 37 °C in a 5% CO_2_ atmosphere, and the culture medium was changed every 2 days.

### cDNA cloning

The coding sequence of SLC7A8 cDNA (encoding 535 amino acids, NM_012244.4) was amplified from the previously established pcDNA3.1-LAT2 vector [19] and subcloned into the pEGFP-C2 vector and named wild-type (WT) hLAT2 plasmid. Correct sequences were confirmed prior to use.

### Site-directed mutagenesis

Predictions of putative PKC phosphorylation sites were conducted using the in silico analysis tool “ScanProsite (https://prosite.expasy.org/scanprosite/)” [20]. The transmembrane regions of hLAT2 protein were predicted using the in silico analysis tool “TMHMM-2.0 (https://services.healthtech.dtu.dk/service.php?TMHMM-2.0)” [21]. Alignment of LAT2 amino acid sequences from five different mammals was performed using the in silico analysis tool “Multialin (http://multalin.toulouse.inra.fr/multalin/)” [22]. Considering the results of those analyses, three sites (Thr-11, Ser-337 and Ser-487) were chosen as targets for the mutagenesis. Site-directed mutagenesis was performed to mutate those three sites in the WT hLAT2 plasmid using PrimeSTAR Max DNA polymerase (TaKaRa, Shiga, Japan). All of the residues were changed to alanine residues using oligonucleotides shown in Additional file 1, and the plasmid was named Triple mut hLAT2 plasmid. The presence of the mutations was verified prior to use.

### Preparation of transient hLAT2 expression system

Parental S2 cells were seeded (5 × 10^5^ cells/mL) and cultured for 24 h, followed by transfection with the WT hLAT2 plasmid or Triple mut hLAT2 plasmid using Lipofectamine 3000 (Thermo Fisher Scientific) according to the manufacturer’s protocol. Parental S2 cells were used as a negative control.

### Treatment with PKC modulators

A broad-spectrum PKC activator, phorbol 12-myristate 13-acetate (PMA) (Nacalai Tesque), and a pan-PKC inhibitor, Go6983 (Tocris Bioscience, Bristol, UK), were used to analyze the effect of PKC on hLAT2.

Parental S2 cells, S2-Mock cells and S2-LAT2 cells were seeded (5 × 10^5^ cells/mL) and cultured for 48 h. To analyze the effect of PMA, the cells were treated with PMA (0.1 or 1 µM contained in the growth medium) or its solvent (0.1% (v/v) dimethyl sulfoxide (DMSO)) for 30 min when 48 h had passed after the seeding. To determine the effect of Go6983 on the PMA-induced effect on hLAT2 activity, the cells were exposed to Go6983 (10 µM) or its solvent (0.1% (v/v) DMSO) at the same timing as the PMA treatment.

For the transient hLAT2 expression system, treatment was started 24 h after the transfection, and the cells were exposed to PMA (1 µM) or its solvent (0.1% (v/v) DMSO) for 1 h.

For Caco-2 cells, the cells were seeded (2 × 10^5^ cells/mL) and cultured for 48 h, and treatment with PMA (0.1 or 1 µM) and/or Go6983 (1 µM) was conducted for 4 h in the same way explained above.

### Transport assay

As a representative substrate of LAT2, [2, 3-^3^H]-labeled L-alanine (alanine) (Moravek Biochemicals, Brea, CA) and non-radiolabeled alanine (Nacalai Tesque) were used for evaluating LAT2 transport activity. 2-Aminobicyclo [2.2.1] heptane-2-carboxylic acid (BCH) (Sigma-Aldrich) and JPH203 (MedKoo Biosciences Inc., Morrisville, NC) were also used as an inhibitor for LAT family transporters and as an LAT1-specific inhibitor, respectively.

After the cells had been washed twice with pre-warmed Na^+^-free Hanks’ balanced salt solution (Na^+^-free HBSS, consisting of 125 mM choline chloride, 4.8 mM KCl, 25 mM HEPES, 1.2 mM KH_2_PO_4_, 1.2 mM MgSO_4,_ 1.3 mM CaCl_2_ and 5.6 mM glucose, pH 7.4), the cells were incubated with Na^+^-free HBSS buffer (250 µL/well) containing alanine (consisting of 1.35 µCi/mL [^3^H]alanine and non-radiolabeled alanine). To inhibit LAT family or LAT1-related uptake activity, BCH (1 mM) or JPH203 (10 µM) was added to the substrate solution, and its solvent, sterilized water (1% (v/v)) or DMSO (0.1% (v/v)), was utilized as its control. The concentration of alanine and incubation time are stated in each figure legend. To stop the incubation, the substrate solution was removed from each well and the cells were washed three times with ice-cold Na^+^-free HBSS buffer containing 1 M non-radiolabeled alanine, followed by solubilization with 0.1 N NaOH (500 µL/well). Solvents were transferred into scintillation vials and the radioactivity was measured by a liquid scintillation analyzer (Tri-Carb 2800TR, PerkinElmer, Tokyo, Japan). Quantification of protein was conducted by the method of BCA using bovine serum albumin (BSA) as a standard.

In kinetic analysis, transport activity levels were calculated by subtracting the value obtained from S2-Mock cells from the value obtained from S2-LAT2 cells. Alanine concentrations used in this analysis were 1, 100, 200, 400, 1000 and 2000 µM, containing [^3^H]alanine (1.35 µCi/mL) and non-radiolabeled alanine. *Km* and *Vmax* values were estimated by carrying out Michaelis–Menten-type non-linear curve fitting using a computer program (Prism7 Ver 7.02, SPSS Inc., Chicago, IL).

### Quantitative real-time PCR analysis

Total RNA was isolated from the cells using Nucleospin RNA plus (MACHEREY–NAGEL, Düren, Germany) according to the manufacturer’s protocol. cDNA was synthesized from the extracted RNA (1 μg) using ReverTra Ace® qPCR RT Master Mix (TOYOBO, Osaka, Japan) according to the manufacturer’s protocol. Quantitative real-time PCR (qPCR) analyses were performed using the Eco Real-Time PCR System (Illumina, San Diego, CA). Quantification of human LAT1 (hLAT1), human LAT2 (hLAT2), mouse glyceraldehyde-3-phosphate dehydrogenase (mGapdh) and human glyceraldehyde-3-phosphate dehydrogenase (hGAPDH) mRNA expression levels was performed using THUNDERBIRD® SYBR® qPCR Mix Kit (TOYOBO) with primer sets (hLAT1 F/R, hLAT2 F1949/R2026, mGapdh 286F/460R and hGAPDH 83F/130R). The mRNA expression levels of mGapdh and hGAPDH were used as internal standards to normalize the hLAT1 and hLAT2 mRNA expression levels. The hLAT1 and hLAT2 mRNA expression levels were expressed as 2^−ΔCT^. All primer sequences used are shown in Additional file 2.

### Protein extraction

Whole cell lysates were prepared from each cell line using RIPA buffer (Nacalai Tesque) supplemented with 1% (v/v) protease inhibitor cocktail (Nacalai Tesque) and 1% (v/v) phosphatase inhibitor cocktail (Nacalai Tesque). After removing cellular debris in the lysate by centrifugation, the supernatants were used in Western blot analysis and Phos-tag SDS-PAGE.

Membrane protein samples were prepared with ultracentrifugation method. First, whole-cell lysates were centrifuged at 1000*g* for 10 min at 4 °C, and the supernatant was then subjected to ultracentrifugation (100,000*g* for 40 min at 4 °C). The supernatant was collected as cytosol fraction. The pellet was solubilized with RIPA buffer, followed by a second ultracentrifugation (100,000*g* for 40 min at 4 °C), and the supernatant was collected as soluble membrane fraction.

### Western blot analysis

The lysates were mixed with 2× laemmli sample buffer (BIORAD, Richmond, CA) supplemented with 5% (v/v) 2-mercaptethanol (2-ME, Nacalai Tesque) in an equal amount and pre-incubated at 37 °C for 30 min. The proteins were separated on a 12.5% SDS–polyacrylamide gel (Anatech, Tokyo, Japan) with a running buffer (25 mM Tris, 192 mM glycine, 1 g/L SDS) and electro-transferred onto a polyvinylidene difluoride membrane (Sigma-Aldrich) with a transblotting buffer (25 mM Tris, 192 mM glycine, 15% (v/v) methanol). Following blocking with 3% (w/v) BSA in TBS-T (25 mM Tris, 137 mM NaCl, 2.68 mM KCl, 0.05% (v/v) Tween-20, pH 7.5), the membranes were incubated with primary antibodies diluted with Can Get Signal® Immunoreaction Enhancer Solution 1 (TOYOBO) at 4℃ overnight (LAT2) or at room temperature for 1 h (β-actin and Na^+^/K^+^-ATPase α1). After washing with TBS-T, the blots were incubated with HRP-conjugated IgG antibodies diluted with Can Get Signal® Immunoreaction Enhancer Solution 2 (TOYOBO) for 1 h at room temperature. Immunocomplexes were detected using Clarity MAX Western ECL Substrate (BIORAD) and visualized by LAS-4000 mini (FUJIFILM, Tokyo, Japan). Details of the antibodies used in this study are shown in Additional file 3.

### Fluorescence analysis

S2 cells were seeded on a collagen-coated cover glass. Transfection and PMA treatment was conducted as described above, and the cells were fixed with 4% (v/v) paraformaldehyde (Nacalai Tesque). Nuclear counter-staining was conducted with DAPI contained in Fluoro-KEEPER Antifade Reagent (Nacalai Tesque) and fluorescence was analyzed using confocal laser scanning immunofluorescence microscopy (FLUOVIEW FV10i, Olympus, Tokyo, Japan) with a 60 × /3 objective. Images were processed with Image J v1.53c (National Institutes of Health, Bethesda, MD).

### Phos-tag SDS-PAGE

The lysates were purified with trichloroacetic acid precipitation to remove EDTA and solubilized with the sample buffer (BIORAD). 2-ME (2.5% (v/v)) and 10 mM ZnCl_2_ (Nacalai Tesque) were added to the purified lysates and the lysates were pre-incubated at 37 °C for 30 min. Phos-tag SDS-PAGE was performed with a running buffer (0.5 M Tris base, 0.5 M MOPS and 0.5% (w/v) SDS) and 8% polyacrylamide gels containing 50 µM Phos-tag acrylamide (FUJIFILM Wako Pure Chemical Corp.) and 100 µM ZnCl_2_. After electrophoresis, Phos-tag acrylamide gels were washed twice with transblotting buffer (Nacalai Tesque) containing 10 mM EDTA for 20 min with gentle shaking and then washed once with transblotting buffer for 10 min. The procedure from the transblotting step was the same as that for Western blotting. To confirm that the Phos-tag SDS-PAGE is working correctly, a positive control sample that contains phosphorylated and dephosphorylated α-casein was purchased from FUJIFILM.

### Coomassie Brilliant Blue (CBB) staining

CBB staining was performed using CBB Stain One Super (Nacalai Tesque) according to the manufacturer’s protocol.

### Cell viability assay

Cell viability was evaluated by the WST-8 assay. Briefly, the cells were seeded into96-well plates and treated with Go6983 (Tocris Bioscience) (1 or 10 µM) or its vehicle (DMSO, 0.1% (v/v)) for incubation periods stated in the figure legend. Cell Count Reagent SF (Nacalai Tesque) was added to each well, after which the plate was incubated at 37 °C for 2 h. Absorbance of the resultant formazan product was measured at 450 nm by a SYNERGY2 microplate reader (BioTek, Winooski, VT). Cell viability was calculated relative to DMSO-treated cells.

### Statistical analysis

Statistical analysis was performed using Statcel 4 software (OMS Publishing Inc., Tokyo, Japan). One-way analysis of variance (ANOVA) was performed for comparison between multiple values. If the ANOVA showed significant differences, a Tukey post hoc test was performed in order to identify significant differences between the values. Unpaired Student’s t-test was performed for comparisons between two values. All data are presented as means ± SD, and the means were compared, with *p* < 0.05 considered significant.

## Results

**Fig. 1:**
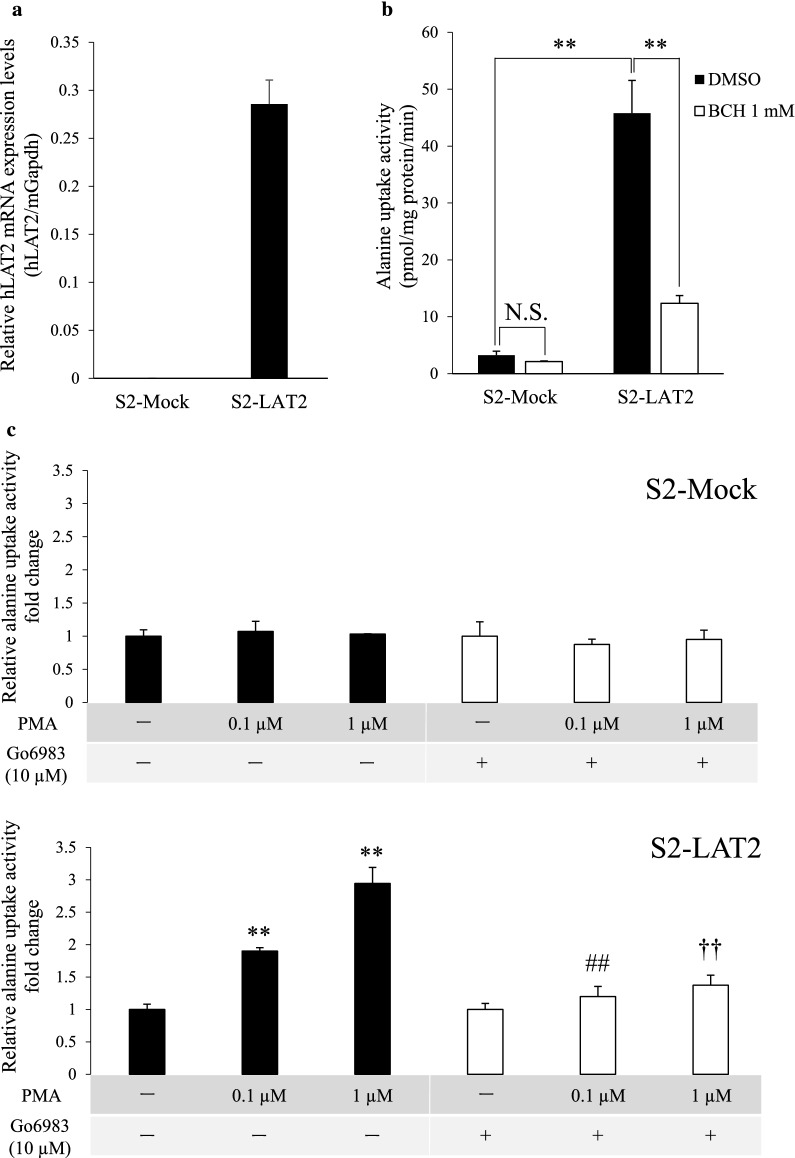
Effect of PKC activation on hLAT2 transport activity in S2-Mock and S2-LAT2 cells. **a** hLAT2 mRNA expression levels in S2-Mock and S2-LAT2 cells were examined by qPCR. The values were normalized to the mGapdh mRNA expression level, and the data are shown as the mean ± SD of values obtained from three separate experiments, each performed in duplicate. **b** Functional expression of hLAT2 in S2-Mock and S2-LAT2 cells was examined by a transport assay. The cells were incubated with alanine (1 µM) in Na^+^-free HBSS buffer for 1 min at 37℃. BCH (1 mM) was used as a LAT inhibitor, and its solvent (0.1% (v/v) DMSO) was used as a control. Data are shown as the mean ± SD of values obtained from three separate experiments, each performed in duplicate. **, *p* < 0.01; NS, not significant. **c** The effect of PKC activation on hLAT2 transport activity in S2-Mock and S2-LAT2 cells was examined by a transport assay. To examine the response of hLAT2 to PKC activation, the cells were treated in the absence (0.1% (v/v) DMSO, -) or presence of PMA (0.1 or 1 µM) for 30 min. To determine whether the PMA-induced effect depends on PKC activation, the cells were exposed to a pan-PKC inhibitor, Go6983 (10 µM), or its solvent (0.1% (v/v) DMSO, -) for 30 min at the same timing as the PMA treatment. After removing the reagents, the cells were incubated with alanine (1 µM) in Na^+^-free HBSS buffer for 1 min at 37℃. Data are shown as the mean ± SD of values obtained from three separate experiments, each performed in duplicate. **, *p* < 0.01 vs -/- (PMA/Go6983), ##, *p* < 0.01 vs 0.1 µM/-, ††, *p* < 0.01 vs 1 µM/-

As the first step to examine the possibility of hLAT2 transport activity being controlled by PKC, we used a stable hLAT2 expression system, S2-Mock and S2-LAT2 cells, for the study. hLAT2 mRNA expression was highly detected in S2-LAT2 cells, and alanine uptake activity in S2-LAT2 cells was significantly higher than that in S2-Mock cells and was significantly inhibited by BCH (Fig. 1a, b). After confirming the hLAT2 functional expression in the cells, we performed a transport assay using cells treated with a PKC activator, PMA, and/or a PKC inhibitor, Go6983. The results showed that alanine uptake activity in S2-LAT2 cells, but not that in S2-Mock cells, was upregulated by PMA (0.1 or 1 µM) treatment in a PMA concentration-dependent manner (Fig. 1c). Moreover, co-treatment with Go6983 (10 µM) significantly prevented the PMA-induced upregulation of hLAT2 transport activity (Fig. 1c), confirming that the PMA effect on hLAT2 was related to PKC activation. A cytotoxicity assay also clarified that Go6983 simply inhibited PKC activation and had no effect on cell viability (Additional file 4).

**Fig. 2:**
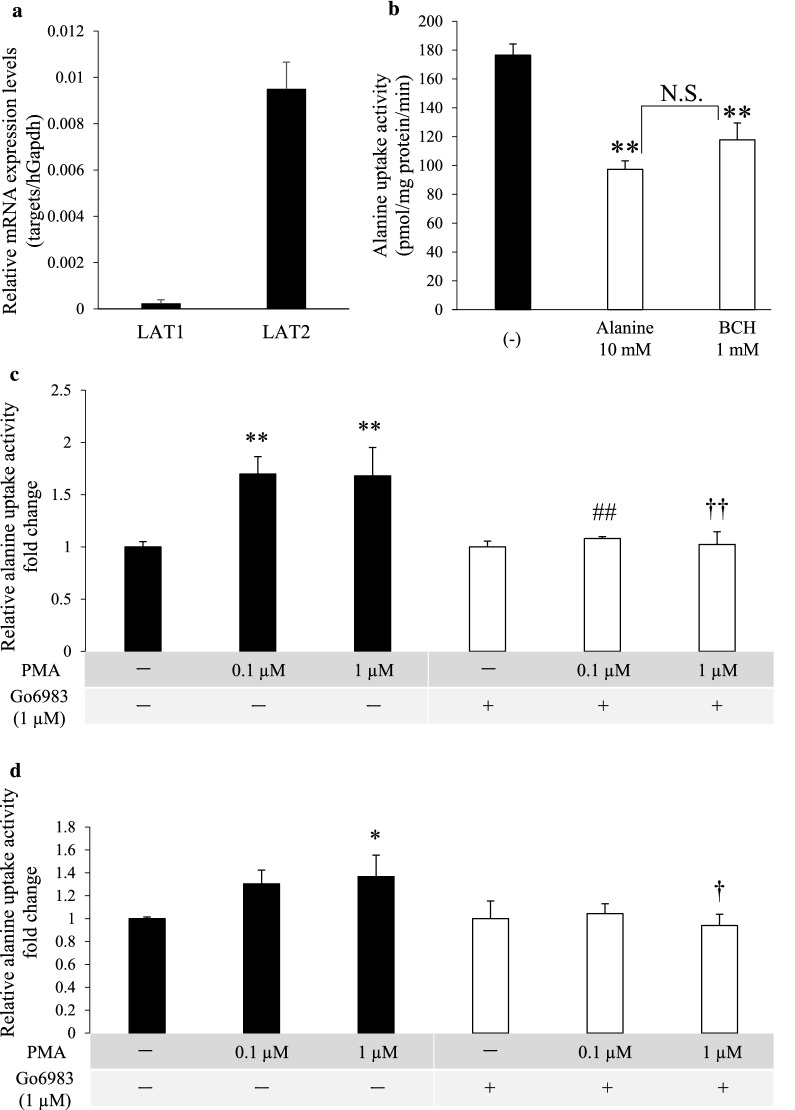
Effect of PKC activation on alanine uptake activity in Caco-2 cells. **a** hLAT1 and hLAT2 mRNA expression levels in Caco-2 cells were examined by qPCR. The values were normalized to the hGAPDH mRNA expression level, and the data are shown as the mean ± SD of values obtained from three separate experiments, each performed in duplicate. **b** Functional expression of hLATs in Caco-2 cells was examined by a transport assay. The cells were incubated with alanine (50 µM) in Na^+^-free HBSS buffer for 5 min at 37℃. Overloaded alanine (10 mM) and BCH (1 mM) were used as inhibitors for alanine uptake-related transporters and LATs, respectively. Its solvent (0.1% (v/v) sterilized water) was used as a control (-). Data are shown as the mean ± SD of values obtained from three separate experiments, each performed in duplicate. **, *p* < 0.01 vs control (-); NS, not significant. **c**, **d** The effect of PKC activation on alanine uptake activity in Caco-2 cells was examined by a transport assay. To activate PKC, the cells were treated in the absence (0.1% (v/v) DMSO, -) or presence of PMA (0.1 or 1 µM) for 4 h. To determine whether the PMA-induced effect depends on PKC activation, the cells were exposed to Go6983 (1 µM) or its solvent (0.1% (v/v) DMSO, -) at the same timing as the PMA treatment. After removing the reagents, alanine uptake was conducted in the same way as explained in **b**. **d** To elucidate the alanine uptake activity by hLAT1, an LAT1-specific inhibitor, JPH203 (10 µM), was added to the substrate solution. Data are shown as the mean ± SD of values obtained from three separate experiments, each performed in duplicate. *, *p* < 0.05; **, *p* < 0.01 vs -/- (PMA/Go6983), ##, *p* < 0.01 vs 0.1 µM/-, †, *p* < 0.05; ††, *p* < 0.01 vs 1 µM/-

Next, we sought to examine the effect of PKC activation on endogenous hLAT2. As a candidate to be used as a tool for the experiments, the human colorectal cancer cell line Caco-2 was chosen, and hLAT1 and hLAT2 mRNA expression levels were first examined. Caco-2 cells showed higher mRNA expression level of hLAT2 than that of hLAT1 (Fig. 2a). The transport assay showed that both overloaded alanine (10 mM) and the LAT inhibitor BCH (1 mM) significantly inhibited alanine uptake activity, almost to the same levels (Fig. 2b), indicating that LAT family plays a major role in alanine uptake in Caco-2 cells. Considering Caco-2 cells to be suitable for the present study, we performed a transport assay using cells treated with PMA (1 µM) and/or Go6983 (1 µM). Alanine uptake activity in Caco-2 cells was significantly upregulated by PMA treatment, and the upregulation was cancelled by co-treatment with Go6983 (Fig. 2c). Furthermore, the upregulation of alanine uptake activity was retained in the presence of the LAT1-specific inhibitor JPH203 (Fig. 2d), indirectly indicating a response of hLAT2 to PKC activation.

**Fig. 3:**
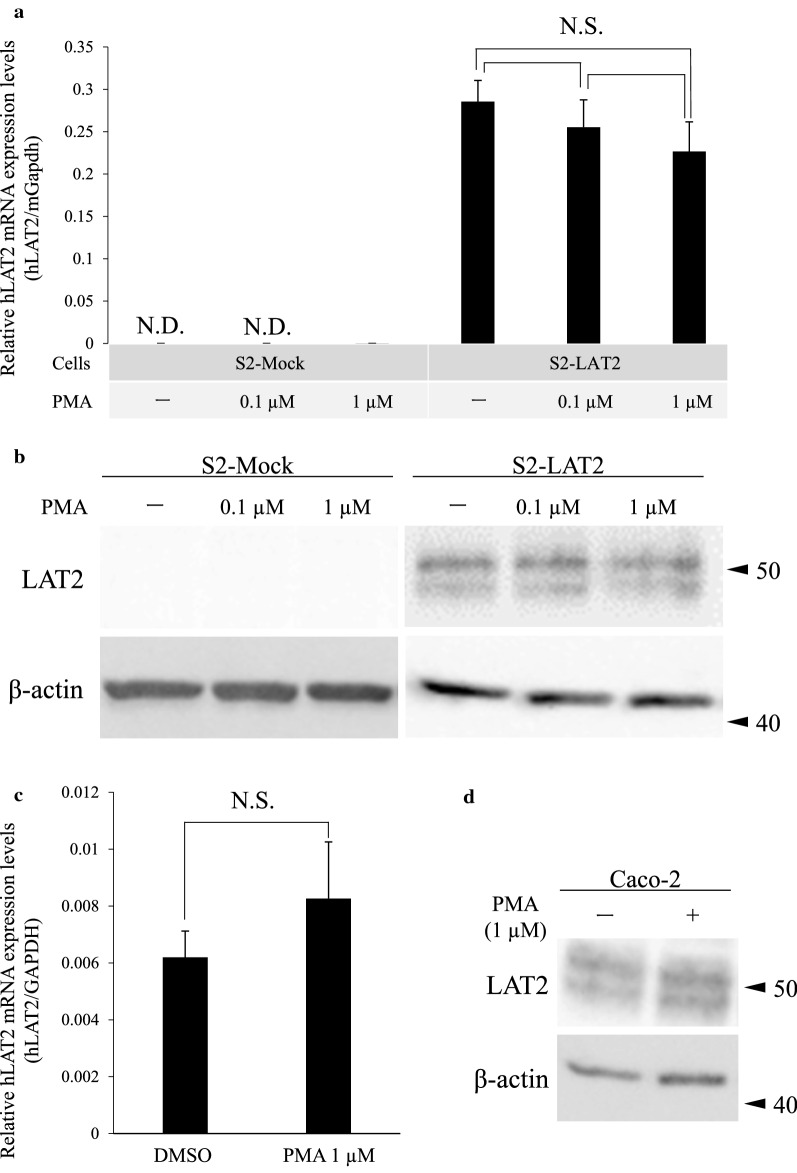
Effect of PKC activation on hLAT2 mRNA and protein expression levels. **a** The effect of PKC activation on hLAT2 mRNA expression levels in S2-Mock and S2-LAT2 cells was examined by qPCR. Total RNA was isolated from the cells that had been treated with DMSO (0.1% (v/v), -) or PMA (0.1 or 1 µM) for 30 min. The values were normalized to the mGapdh mRNA expression level, and the data are shown as the mean ± SD of values obtained from three separate experiments, each performed in duplicate. NS, not significant. **b** The effect of PKC activation on hLAT2 protein expression levels in S2-Mock and S2-LAT2 cells was examined by Western blot analysis. Whole cell lysates were prepared from cells that had been treated in the same way as explained in **a**. β-Actin was used as a loading control, and representative results that were obtained from three independent experiments are shown. **c** The effect of PKC activation on hLAT2 mRNA expression levels in Caco-2 cells was examined by qPCR. Total RNA was isolated from the cells that had been treated with DMSO (0.1% (v/v)) or PMA (1 µM) for 4 h. The values were normalized to the hGAPDH mRNA expression level, and the data are shown as the mean ± SD of values obtained from three separate experiments, each performed in duplicate. NS, not significant. **d** The effect of PKC activation on hLAT2 protein expression levels in Caco-2 cells was examined by Western blot analysis. Whole cell lysates were prepared from cells that had been treated in the same way as explained in **c**. β-Actin was used as a loading control, and representative results that were obtained from three independent experiments are shown

To determine whether PKC activation has any effect on de novo synthesis of hLAT2, qPCR and Western blot analyses were performed using S2-Mock, S2-LAT2 and Caco-2 cells. The results of qPCR analysis showed that the mRNA expression level of hLAT2 was not affected by PMA (0.1 or 1 µM) treatment in all of the cells examined (Fig. 3a, c). Likewise, Western blot analysis showed that hLAT2 protein expression levels were not different in cells treated with DMSO (0.1% (v/v)) and cells treated with PMA (0.1 or 1 µM) at the whole cell lysate level (Fig. 3b, d).

**Fig. 4:**
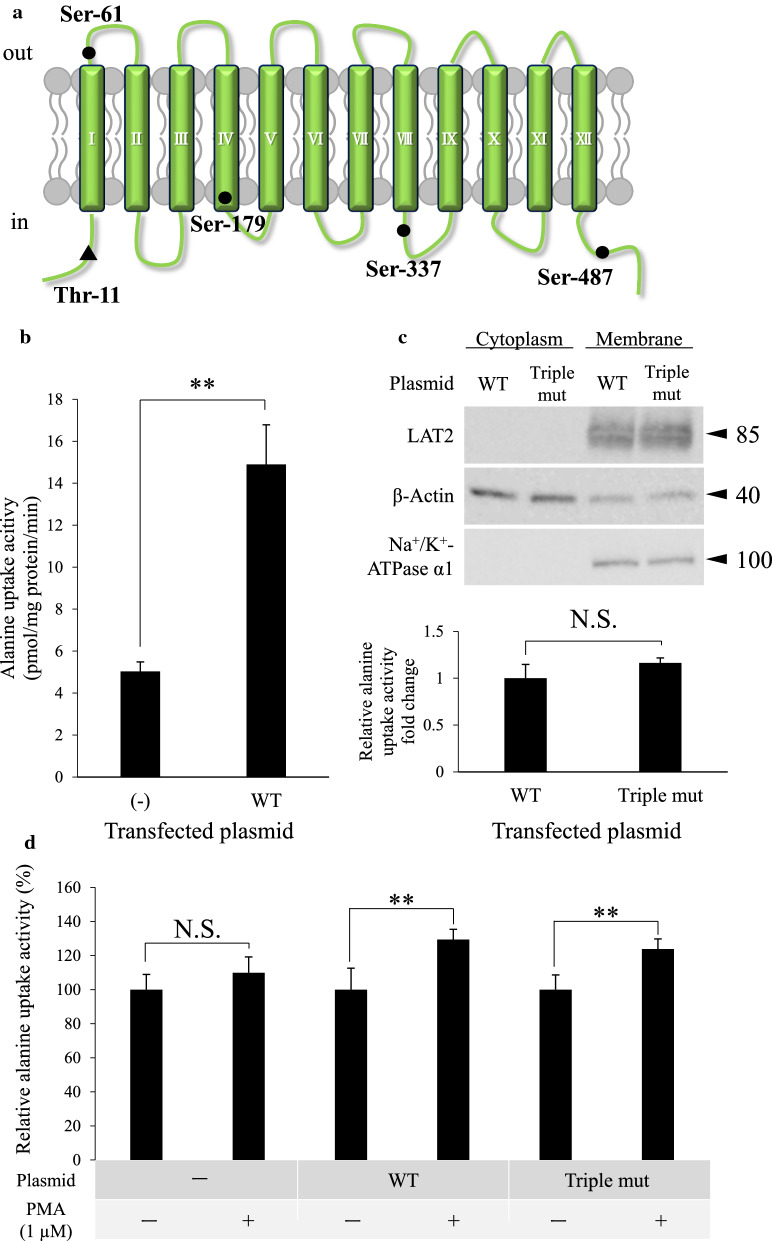
Effect of putative PKC phosphorylation sites on hLAT2 transport activity. **a** Five putative PKC phosphorylation sites in hLAT2 protein are shown. The putative PKC phosphorylation sites and the transmembrane regions in hLAT2 protein were predicted by the in silico analysis tools “ScanProsite” and “TMHMM-2.0”, respectively. **b**, **c** Alanine uptake activity and hLAT2 protein expression levels at cell membrane in a transient hLAT2 expression system were examined by a transport assay and Western blot analysis, respectively. S2 cells were transfected with WT hLAT2 (WT) or Triple mut hLAT2 (Triple mut) plasmid, and non-transfected S2 cells were used as a negative control (-). In transport assay, the cells were incubated with alanine (1 µM) in Na^+^-free HBSS buffer for 2 min at 37℃. Data are shown as the mean ± SD of values obtained from four (**b**) or three (**c**) separate experiments, each performed in duplicate. **, *p* < 0.01; NS, not significant. Western blot analysis was performed using protein samples prepared with ultracentrifugation method. β-actin and Na^+^/K^+^-ATPase α1 were both used as loading controls and also used as a cytosol marker and a cell membrane marker, respectively. Representative results that were obtained from three independent experiments are shown. **d** The effect of PKC activation on WT and Triple mut hLAT2 transport activity was examined by a transport assay. Cells were prepared in the same way as explained in **b**, **c**, and the cells were treated with DMSO (0.1% (v/v), -) or PMA (1 µM) for 1 h. After removing the reagents, the cells were incubated with alanine (1 µM) in Na^+^-free HBSS buffer for 2 min at 37℃. Data are shown as the mean ± SD of values obtained from four separate experiments, each performed in duplicate. **, *p* < 0.01; NS, not significant

We next investigated whether the PMA-induced upregulation of hLAT2 function was attributable to PKC-dependent phosphorylation of hLAT2 itself. As shown in Fig. 4a, in silico analysis showed that five serine or threonine residues (Thr-11, Ser-61, Ser-179, Ser-337 and Ser-487) are strong candidates for phosphorylation by PKC, and three of them (Thr-11, Ser-337 and Ser-487) were predicted to be located at the intracellular loops of hLAT2. Moreover, alignment of LAT2 amino acid sequences from five different mammals showed that those sites are retained among the five different mammals (Additional file 5), indicating the importance of those sites. Therefore, we focused on the three potential PKC phosphorylation sites and examined whether those sites are related to the upregulation of hLAT2 function by PKC activation. We constructed plasmids containing WT hLAT2 CDS or hLAT2 CDS for which the three potential PKC phosphorylation sites were mutated to alanine residues (Triple mut). Plasmids also contain EGFP CDS next to N-terminal of the hLAT2 CDS, which makes it possible to analyze the localization of hLAT2 in transfected S2 cells. First, alanine uptake activity of WT or Triple mut hLAT2 was examined by a transport assay to confirm that the transient WT hLAT2 expression system worked well (Fig. 4b). Localization of both WT and Triple mut hLAT2 proteins at cell membrane of S2 cells was also confirmed with confocal microscopy analysis (Additional file 6). However, there were no significant differences between the protein expression levels and the alanine uptake activity of WT and Triple mut hLAT2 (Fig. 4c). Moreover, by examining the responses of WT and Triple mut hLAT2 to PMA (1 µM) treatment, it was clarified that there was no difference in the responses of WT and Triple mut hLAT2, in both of which hLAT2 showed upregulated uptake activity with PMA treatment (Fig. 4d). To obtain a clue as to whether hLAT2 protein is phosphorylated by PKC or not, we conducted Phos-tag SDS-PAGE for whole cell lysates of S2-LAT2 cells treated with PMA (0.1 or 1 µM). As a result, the band of hLAT2 did not shift, indicating that hLAT2 phosphorylation by PKC is unlikely (Additional file 7).

**Fig. 5:**
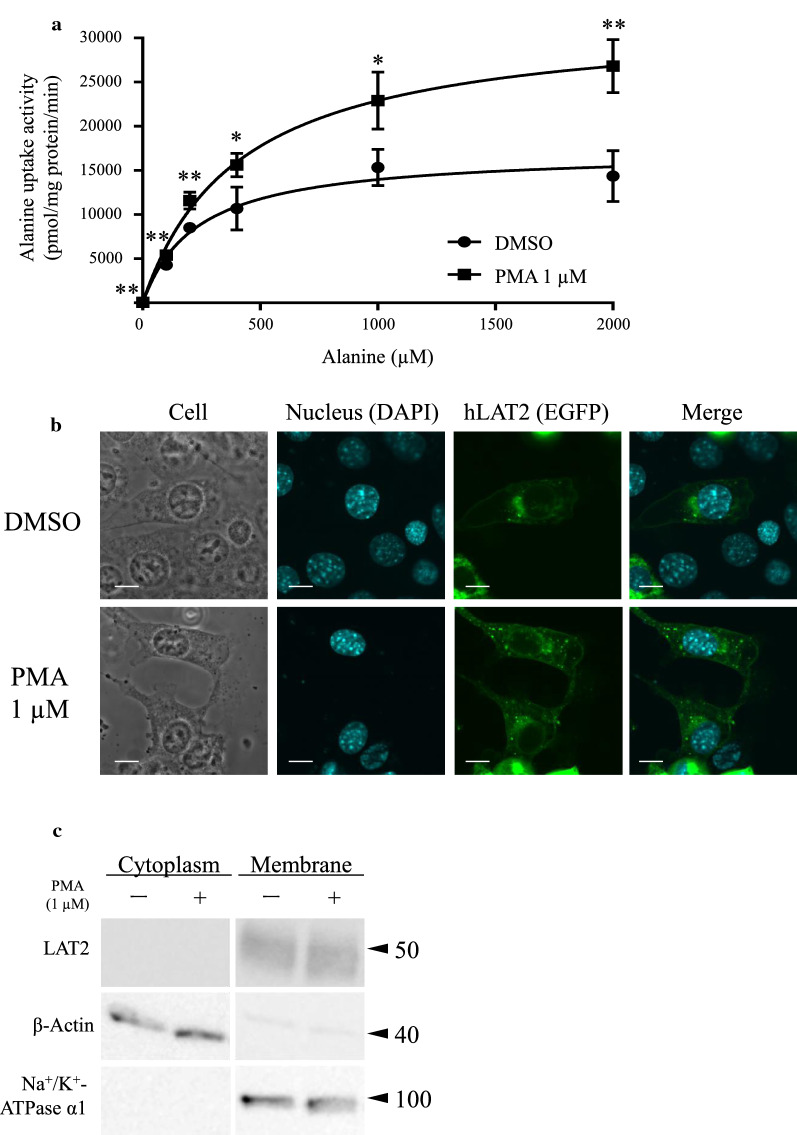
Effect of PKC activation on hLAT2 localization at cell membrane. **a** The effect of PKC activation on the transport kinetics of hLAT2-mediated alanine uptake was examined by a kinetic analysis. S2-Mock cells and S2-LAT2 cells were treated with DMSO (0.1% (v/v)) or PMA (1 µM) for 30 min. Pre-treated cells were incubated with alanine (1, 100, 200, 400, 1000 and 2000 µM) in Na^+^-free HBSS buffer for 1 min at 37℃. Transport activity levels were calculated by subtracting the value obtained from S2-Mock cells from the value obtained from S2-LAT2 cells. Kinetic parameters were estimated by carrying out Michaelis–Menten-type non-linear curve fitting*.* Data are shown as the mean ± SD of values obtained from three separate experiments, each performed in duplicate. *, *p* < 0.05; **, *p* < 0.01 vs DMSO; NS, not significant. **b** The effect of PKC activation on hLAT2 protein localization was examined by confocal microscopy analysis. S2 cells transfected with WT hLAT2 plasmid were treated with DMSO (0.1% (v/v)) or PMA (1 µM) for 1 h. After removing the reagents, cells were fixed with 4% paraformaldehyde and the localization of EGFP-tagged WT hLAT2 protein (green) was examined by confocal microscopy. DAPI (cyan) was used for nuclear counter-staining. Experiments were repeated three times and representative images are shown. Scale bar, 10 µm. **c** The effect of PKC activation on hLAT2 protein expression levels at cell membrane was examined by Western blot analysis. S2-LAT2 cells were treated with DMSO (0.1% (v/v)) or PMA (1 µM) for 30 min, and protein samples were prepared with ultracentrifugation method. β-Actin and Na^+^/K^+^-ATPase α1 were both used as loading controls and also used as a cytosol marker and a cell membrane marker, respectively. Representative results that were obtained from three independent experiments are shown

Finally, we investigated whether the PMA-induced upregulation of hLAT2 function was induced by membrane insertion of hLAT2. First, we performed a kinetic analysis using six different concentrations of alanine (1, 100, 200, 400, 1000 and 2000 µM). As a result, alanine uptake activity was significantly upregulated by PMA treatment (1 µM) at every alanine concentration. Moreover, Michaelis–Menten-type non-linear curve fitting showed that PMA (1 µM) treatment resulted in a significantly increased *Vmax* value (17,187 ± 1222 pmol/mg protein/min with DMSO (0.1% (v/v))-treated S2-LAT2 cells, and 32,309 ± 1678 pmol/mg protein/min in the presence of PMA, *p* < 0.01) with no significant change of *Km* value (230.4 ± 55.14 µM with DMSO (0.1% (v/v))-treated S2-LAT2 cells, and 410.9 ± 60.22 µM in the presence of PMA). Considering this result as an indication of PKC-mediated upregulation of hLAT2 protein expression levels at cell membrane, we performed confocal microscopy analysis using transient WT hLAT2 expression system and Western blot analysis using S2-LAT2 cells. However, the results showed that the localization of hLAT2 protein was not significantly changed by PMA (1 µM) treatment (Fig. 5b). Likewise, Western blot analysis showed that hLAT2 protein expression levels at cell membrane were not different in cells treated with DMSO (0.1% (v/v)) and cells treated with PMA (1 µM) (Fig. 5c).

## Discussion

In the present study, it was clarified that hLAT2 function is upregulated by PKC activation. In most previous studies on the effect of PKC on LAT family, the effect of PKC on the family, not the effect on each member, was investigated. Regarding the effect of PKC on all of the LAT family members, it has been reported that the transport activity of LAT family members was upregulated by PKC activation in human placenta cells, T cells and liver cells [15–17]. Past reports have shown that the responses of transporters to PKC activation can change depending on the organs and species from which the cells are derived [23, 24]. For the LAT family, there have been some reports showing that the family function was not affected by PKC activation [25, 26]. However, the above-mentioned reports indicate the possibility that PKC can stimulate the function of LAT2 as one of the LAT family members and that the phenomenon can also be detected in proximal tubule cells and intestinal cells derived from organs that are the main organs with high expression levels of LAT2. LAT1 is the only LAT family member for which its regulation system by PKC has been examined [16, 27], and our present study is the first study focusing on the effect of PKC on a single LAT family member LAT2. In the previous study using primary proximal tubule cells, the effect of PKC only on the sodium-dependent alanine uptake activity was analyzed, and it was clarified that PKC had no effect on that activity [28]. Therefore, we provide the first information about the effect of PKC on sodium-independent uptake activity of alanine in proximal tubule cells, and we showed that hLAT2 function is stimulated by PKC activation. In the human intestinal cell line Caco-2, it was also shown that sodium-independent and BCH-sensitive alanine uptake activity, which can be considered to be equal to LAT-mediated alanine uptake activity, was upregulated by PKC activation. When interpreting this result, it is important to know that LAT2 has much higher affinity for alanine than do other LAT family members [3, 29, 30]. Although more detailed investigation is needed to certify the relation between hLAT2 and PKC in Caco-2 cells, the relation was partially clarified by our obtained results showing a sustained upward tendency of alanine uptake activity in the presence of the LAT1-specific inhibitor JPH203.

In our present study, we do not know how PKC activation is related to the regulation of hLAT2 function. However, there are three possible ways that PKC can regulate LAT2 function: (1) de novo synthesis of the transporter, (2) improvement of the transporter activity by the phosphorylation itself and (3) inducing the insertion of the transporter from the cytosol to the cell membrane [14]. Regarding the first possibility, we showed that PKC activation by PMA did not have a sufficient impact to modify the total mRNA and protein expression levels, indicating that de novo synthesis was not the main factor for the stimulation of hLAT2 function by PKC. Regarding the second possibility, we considered that PKC can regulate hLAT2 function by phosphorylating hLAT2 itself, since the hLAT2 amino acid sequence is predicted to possess several sites (Thr-11, Ser-337 and Ser-487) that might be phosphorylated by PKC. However, our results showed that none of the three candidates were related to the upregulation of hLAT2 transport activity by PKC activation, indicating that regulation of hLAT2 function by direct phosphorylation by PKC is unlikely. Considering those results, the third possibility was the most likely mechanism for the regulation of hLAT2 function, which was indicated by the PKC-mediated upregulation of *Vmax* value for alanine transport (Fig. 5a). However, contrary to our expectation, it was clarified that hLAT2 membrane insertion was not significantly accelerated by PKC activation (Fig. 5b, c). Considering these results, facilitation of outward-to-inward transition of hLAT2 protein structure could be regarded as one of the possibilities to explain the results. At present, there are no experimental methods to examine the frequency or speed of hLAT2 protein to rearrange its structure to translocate its substrates across itself. Together with that, difficulties to dissect the molecular mechanisms underlying transport, due to the lack of crystal structure information for hLAT2, also make us hard to examine this possibility. However, translocation mechanism of hLAT2 has been gradually clarified using the crystal structure information for several distant bacterial homologs of hLATs [31, 32]. Since some previous studies have shown that there are several LAT2 amino acid residues, which were expected to alter *Vmax* value of LAT2-mediated alanine uptake, it may be valuable to analyze the relation between PKC and such residues in the future study.

Together with the mechanism, it would be interesting to investigate the situation in which the upregulation of LAT2 function through PKC activation occurs. In kidney at where LAT2 is most highly expressed, it has been known that PKC activation plays a role to pass the signal from parathyroid hormone to regulate transport of phosphate for maintaining phosphate homeostasis and for regulating bone metabolism [33]. Similar to that situation, some amino acid sensing pathways may induce PKC activation mediated upregulation of LAT2 function for maintaining amino acid homeostasis. Regarding roles of amino acids, the LAT2 function upregulation may also be induced under the circumstances that requires cell growth, proliferation and development in both normal and abnormal cells. This idea is supported by the results of previous studies showing that well-known hormones that regulate cell proliferation, such as dihydrotestosterone, affected LAT2 function [34, 35] and also affected another transporter via PKC activation [36]. Since there have been few studies about such situations in which the function of LAT2 is modified and about the physiological and pathological roles of LAT2, it is difficult to discuss about this topic at present. Therefore, investigating for such situations and roles would be necessary to deepen our understanding for the relation between PKC and LAT2 in the future.

## Conclusions

In conclusion, we clearly showed that hLAT2 function is upregulated by PKC activation in proximal tubule cells and intestinal cells, which is not related to either the de novo synthesis, the phosphorylation or the membrane insertion of hLAT2. Since LAT2 is known to transport various amino acids and some hormones that play great roles in the human body, we believe that studies to clarify the regulation system of LAT2, including our study, are important to deepen our understanding for the physiological and pathological roles of LAT2 in the human body. Considering that LAT2 also transports L-DOPA, which is known to be related to hypertension [37] and some neurodegenerative diseases [38–40], the results of such studies may also contribute to the establishment of new targets for pathological treatment. Moreover, since PKC is known to be activated in the kidney to pass the signal from angiotensin II and parathyroid hormone to regulate blood pressure and bone metabolism [33, 41], LAT2, whose function is clarified to be regulated by PKC, can also be related to those physiology and thus can serve as a potential drug target in the treatment of hypertensive disease and skeletal disease.

### Supplementary Information

**Additional file 1.** Oligonucleotide primers used for site-direct mutagenesis. Data that show sequences of the oligonucleotide primers used for site-direct mutagenesis in the study.

**Additional file 2.** Oligonucleotide primers used for qPCR in the study. Data that show sequences of the oligonucleotide primers used for qPCR in the study.

A**Additional file 3.** Antibodies used for Western blot analysis. Data that show details of the antibodies used for Western blot analysis in the study.

**Additional file 4.** Influence of a pan-PKC inhibitor, Go6983, on S2-Mock, S2-LAT2 and Caco-2 cell viability. Data that show the influence of a pan-PKC inhibitor, Go6983, on S2-Mock, S2-LAT2 and Caco-2 cell viability.

**Additional file 5.** Alignment of LAT2 amino acid sequences from five different mammals. Data that show the alignment of LAT2 amino acid sequences from five different mammals.

**Additional file 6.** Localization of EGFP-tagged WT hLAT2 or Triple mut hLAT2 protein in S2 cells. Data that show the localization of WT hLAT2 or Triple mut hLAT2 protein in S2 cells.

**Additional file 7.** Effect of PKC activation on the hLAT2 phosphorylation state in S2-LAT2 cells. Data that show the effect of PKC activation on the hLAT2 phosphorylation state in S2-LAT2 cells.

## Data Availability

Not applicable.
