# Supplementary material for: Protein kinase C activation upregulates human L-type amino acid transporter 2 function
Source: J Physiol Sci. 2021 Mar 31;71:11. doi: 10.1186/s12576-021-00795-0 (PMC10716992; doi:10.1186/s12576-021-00795-0)
Supplement: Supplementary file 1 — Additional file 1. Oligonucleotide primers used for site-direct mutagenesis. Data that show sequences of the oligonucleotide primers used for site-direct mutagenesis in the study. [file 12576_2021_795_MOESM1_ESM.doc]

**Supplementary file 1. Oligonucleotide primers used for site-directed mutagenesis**

| **Mutations** | **Primer sequences** | |
| --- | --- | --- |
| hLAT2 T11A | Sense | 5’- AAA CAA CGC CGA AAA GAA ACA CCC AG -3’ |
|  | Antisense | 5’- TTT TCG GCG TTG TTT CGG TGC CTG GC -3’ |
| hLAT2 S337A | Sense | 5’- CTT CAC CGC CTC TCG GCT GTT CTT CG -3’ |
|  | Antisense | 5’- CGA GAG GCG GTG AAG AGA GAC CCA TT -3’ |
| hLAT2 S487A | Sense | 5’- CTG GTG GCC CAG AAG ATG TGT GTG GT -3’ |
|  | Antisense | 5’- CTT CTG GGC CAC CAG GGT TAG CAG CTC -3’ |

LAT2, l-type amino acid transporter 2. Underlined letters indicate mutations.
