# Supplementary material for: Protein kinase C activation upregulates human L-type amino acid transporter 2 function
Source: J Physiol Sci. 2021 Mar 31;71:11. doi: 10.1186/s12576-021-00795-0 (PMC10716992; doi:10.1186/s12576-021-00795-0)
Supplement: Supplementary file 2 — Additional file 2. Oligonucleotide primers used for qPCR in the study. Data that show sequences of the oligonucleotide primers used for qPCR in the study. [file 12576_2021_795_MOESM2_ESM.doc]

**Supplementary file 2. Oligonucleotide primers used for qPCR**

| **Names** | **Sequences** |
| --- | --- |
| hLAT1 F | 5’-TGT ACG TGC TGA CCA ACC TG-3’ |
| hLAT1 R | 5’-ATG ACG CCC AGG TGA TAG TTC-3’ |
| hLAT2 F1949 | 5’-CAC GGT TGC TGG ACA GAT AG -3’ |
| hLAT2 R2026 | 5’-GGG AAC AGC AGG TTG ATC TT-3’ |
| hGAPDH F83 | 5’-AGC CAC ATC GCT CAG ACA C-3’ |
| hGAPDH R130 | 5’-GCC CAA TAC GAC CAA ATC C-3’ |
| mGapdh 286F | 5’-AAG CCC ATC ACC ATC TTC CAG G-3’ |
| mGapdh 460R | 5’-GGT TCA CAC CCA TCA CAA ACA T-3’ |

LAT, l-type amino acid transporter. hGAPDH, human glyceraldehyde-3-phosphate dehydrogenase. mGapdh, mouse glyceraldehyde-3-phosphate dehydrogenase.
