# Supplementary material for: Protein kinase C activation upregulates human L-type amino acid transporter 2 function
Source: J Physiol Sci. 2021 Mar 31;71:11. doi: 10.1186/s12576-021-00795-0 (PMC10716992; doi:10.1186/s12576-021-00795-0)
Supplement: Supplementary file 3 — AAdditional file 3. Antibodies used for Western blot analysis. Data that show details of the antibodies used for Western blot analysis in the study. [file 12576_2021_795_MOESM3_ESM.doc]

**Supplementary file 3.** **Antibodies used for Western blot analysis**

| **Antibody** | **Distributor** | **Dilution** |
| --- | --- | --- |
| Rabbit polyclonal anti-SLC7A8 antibody | MBL | 1:1,000 |
| Mouse monoclonal anti-ß-actin antibody | Santa Cruz | 1:10,000 |
| Mouse monoclonal Na^+^/K^+^-ATPase α1 antibody | Santa Cruz | 1:200 |
| HRP-labeled anti-rabbit IgG antibody | GE Healthcare | 1:20,000 |
| HRP-labeled anti-mouse IgG antibody | GE Healthcare | 1:20,000 |
