# Supplementary material for: Protein kinase C activation upregulates human L-type amino acid transporter 2 function
Source: J Physiol Sci. 2021 Mar 31;71:11. doi: 10.1186/s12576-021-00795-0 (PMC10716992; doi:10.1186/s12576-021-00795-0)
Supplement: Supplementary file 4 — Additional file 4. Influence of a pan-PKC inhibitor, Go6983, on S2-Mock, S2-LAT2 and Caco-2 cell viability. Data that show the influence of a pan-PKC inhibitor, Go6983, on S2-Mock, S2-LAT2 and Caco-2 cell viability. [file 12576_2021_795_MOESM4_ESM.pdf]

Supplementary file 4

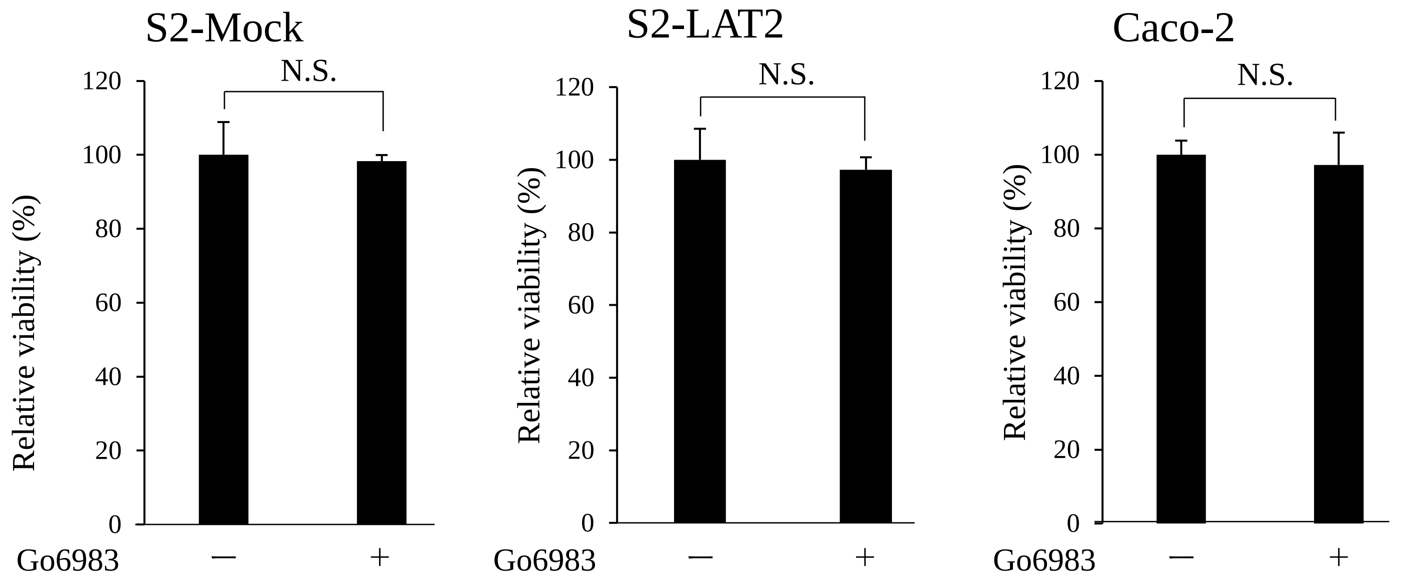

Influence of a pan-PKC inhibitor, Go6983, on S2-Mock, S2-LAT2 and Caco-2 cell viability
The viability of S2-Mock, S2-LAT2 and Caco-2 cells treated with Go6983 was examined by the WST-8 assay. S2-Mock and S2-LAT2 cells were treated with Go6983 (10 µM) for 30 min, and Caco-2 cells were treated with Go6983 (1 µM) for 4 hrs. The cells treated with its solvent (0.1% (v/v) DMSO, -) were used as a control. A cell count reagent was subsequently applied and the cells were incubated for 2 hrs. Data are shown as the mean ± S.D. of values obtained from three separate experiments, each performed in duplicate. N.S., not significant.
