# Supplementary material for: Protein kinase C activation upregulates human L-type amino acid transporter 2 function
Source: J Physiol Sci. 2021 Mar 31;71:11. doi: 10.1186/s12576-021-00795-0 (PMC10716992; doi:10.1186/s12576-021-00795-0)
Supplement: Supplementary file 5 — Additional file 5. Alignment of LAT2 amino acid sequences from five different mammals. Data that show the alignment of LAT2 amino acid sequences from five different mammals. [file 12576_2021_795_MOESM5_ESM.pdf]

Supplementary file 5

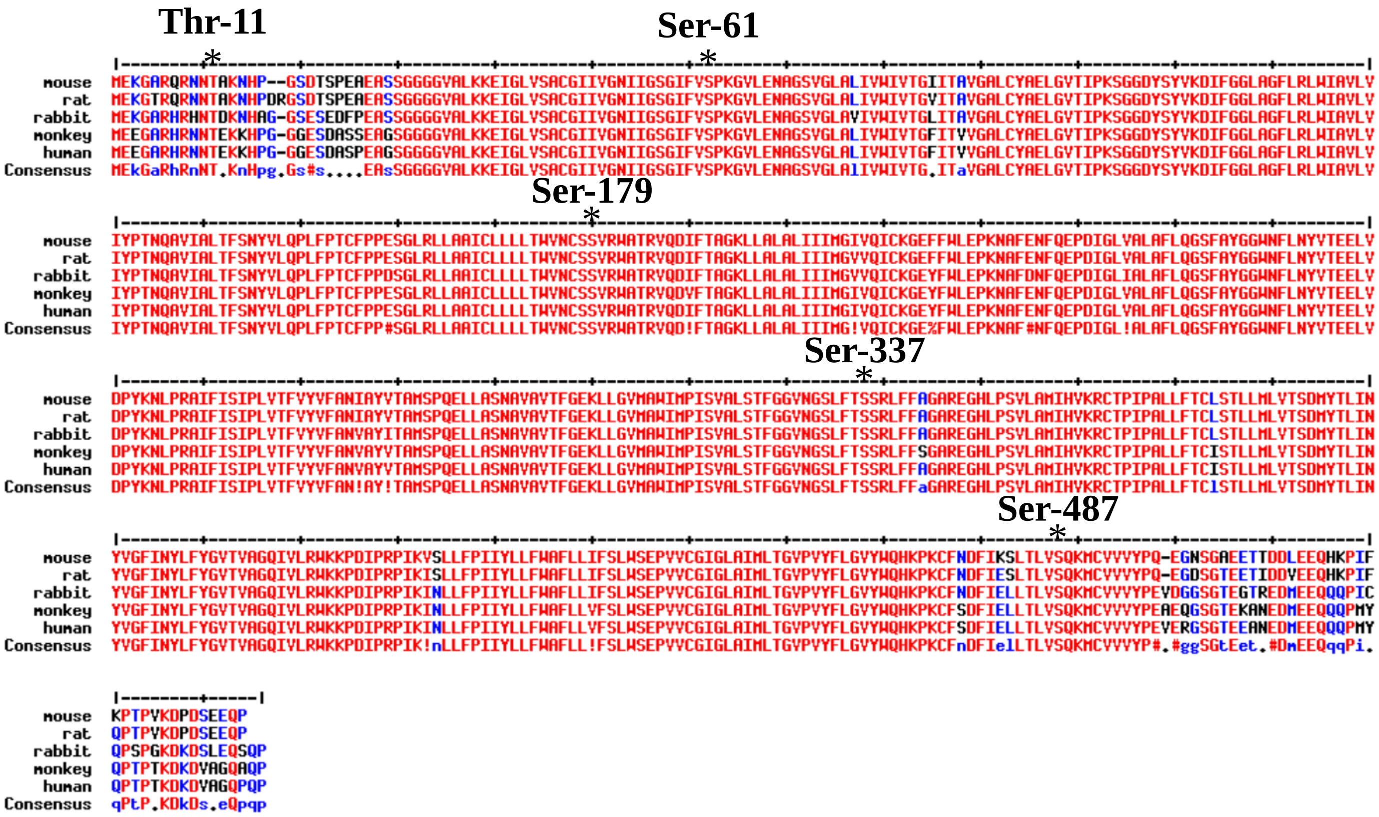

Alignment of LAT2 amino acid sequences from five different mammals

Alignment of LAT2 amino acid sequences from five different mammals (mus musculus (mouse): NM_016972.2, rattus norvegicus (rat): NM_053442.1, oryctolagus cuniculus (rabbit): NM_001082682.1, macaca mulatta (monkey): NM_001257753.1, homo sapiens (human): NM_012244.4) is shown. The five putative PKC phosphorylation sites are indicated by *. Amino acid sequence alignment was performed using the *in silico* analysis tool “Multialin”.
