# Supplementary material for: Protein kinase C activation upregulates human L-type amino acid transporter 2 function
Source: J Physiol Sci. 2021 Mar 31;71:11. doi: 10.1186/s12576-021-00795-0 (PMC10716992; doi:10.1186/s12576-021-00795-0)
Supplement: Supplementary file 6 — Additional file 6. Localization of EGFP-tagged WT hLAT2 or Triple mut hLAT2 protein in S2 cells. Data that show the localization of WT hLAT2 or Triple mut hLAT2 protein in S2 cells. [file 12576_2021_795_MOESM6_ESM.pdf]

Supplementary file 6

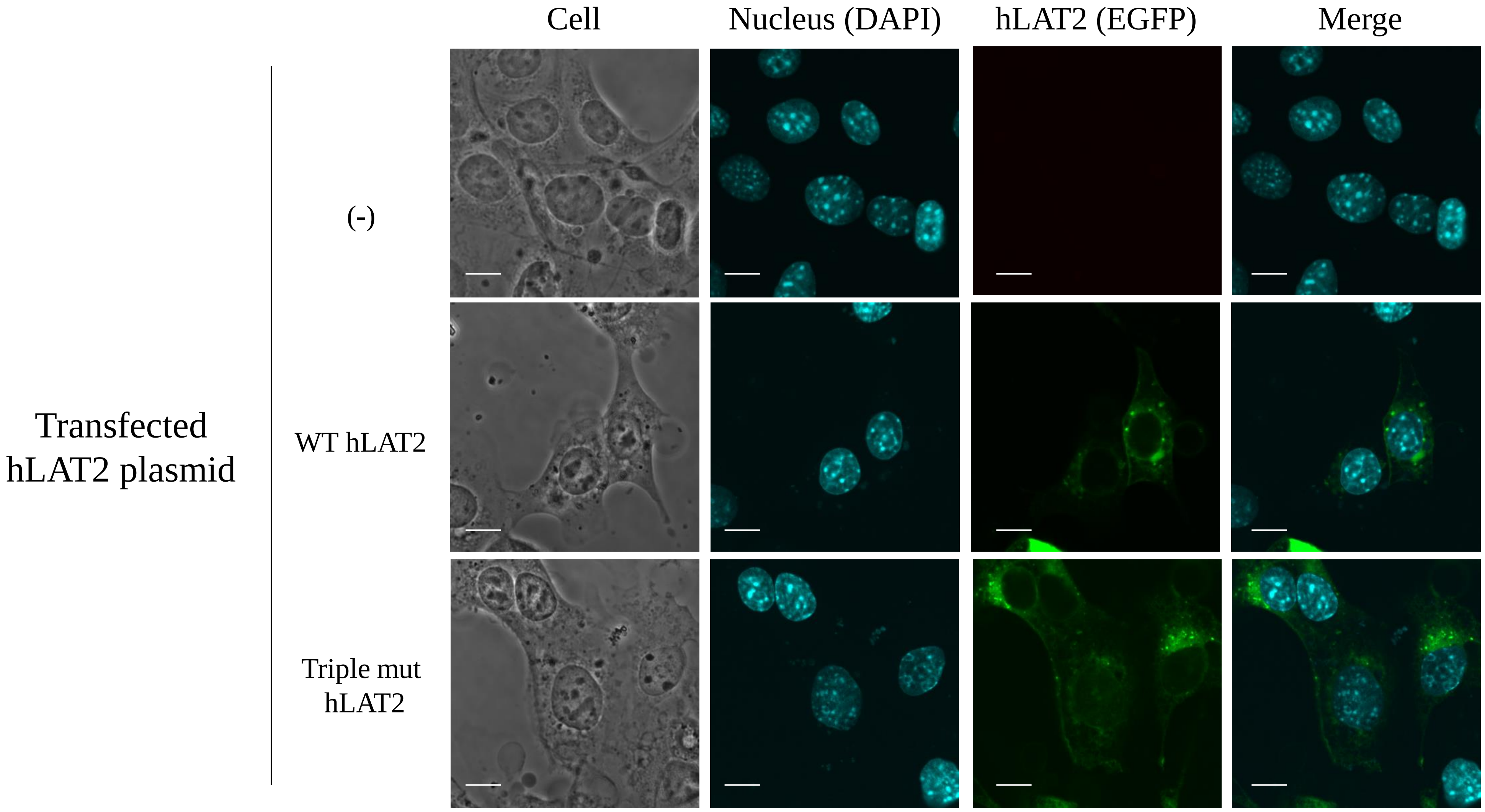

Localization of EGFP-tagged WT hLAT2 or Triple mut hLAT2 protein in S2 cells
S2 cells transfected with WT hLAT2 plasmid or Triple mut hLAT2 plasmid were fixed with 4% PFA, and the localization of each EGFP-tagged hLAT2 protein (green) was examined by confocal microscopy. Non-transfected S2 cells (-) were used as a negative control of hLAT2 protein expression. DAPI (cyan) was used for nuclear counter-staining. Experiments were repeated three times and representative images are shown. Scale bar, 10 μm.
