# Supplementary material for: Protein kinase C activation upregulates human L-type amino acid transporter 2 function
Source: J Physiol Sci. 2021 Mar 31;71:11. doi: 10.1186/s12576-021-00795-0 (PMC10716992; doi:10.1186/s12576-021-00795-0)
Supplement: Supplementary file 7 — Additional file 7. Effect of PKC activation on the hLAT2 phosphorylation state in S2-LAT2 cells. Data that show the effect of PKC activation on the hLAT2 phosphorylation state in S2-LAT2 cells. [file 12576_2021_795_MOESM7_ESM.pdf]

Supplementary file 7

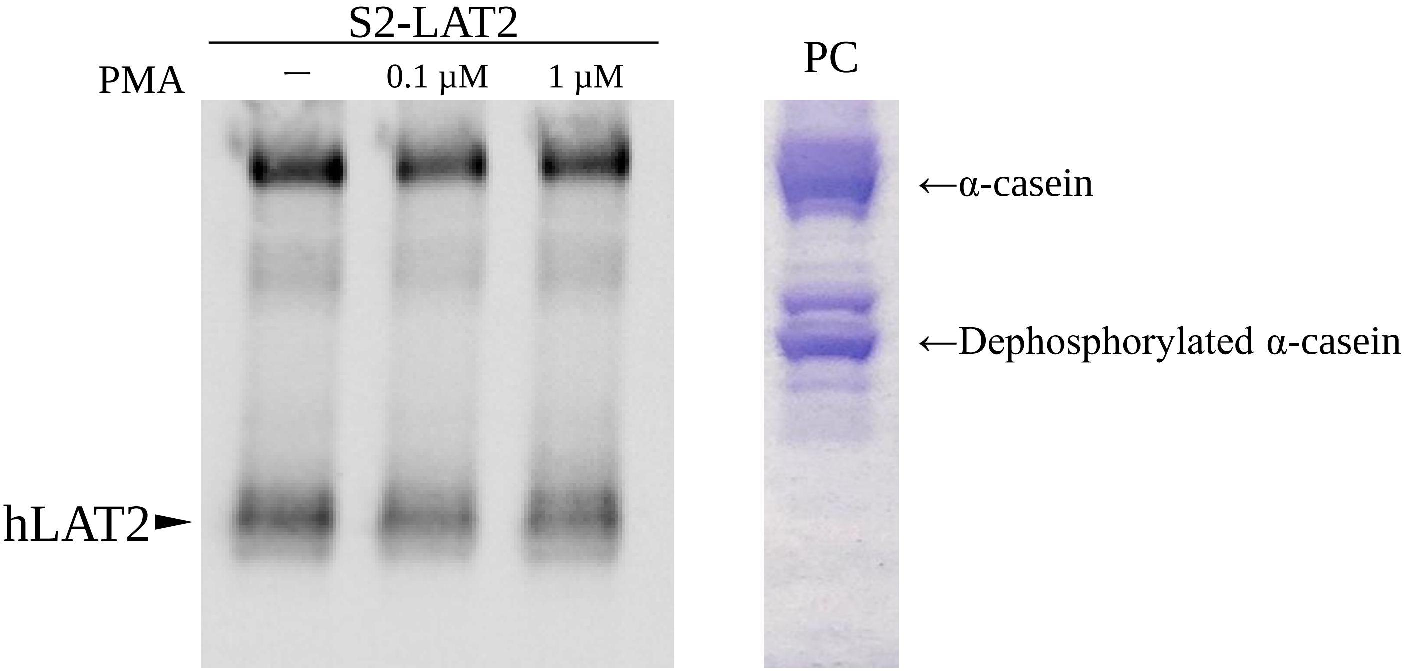

Effect of PKC activation on the hLAT2 phosphorylation state in S2-LAT2 cells

The phosphorylation state of hLAT2 protein in S2-LAT2 cells was examined by Phos-tag SDS-PAGE. Whole cell lysates were prepared from S2-LAT2 cells that had been treated with DMSO (0.1% (v/v), -) or PMA (0.1 or 1 µM) for 30 min. The lysates were separated on polyacrylamide gels containing Phos-tag acrylamide (50 µM) and $ZnCl_2$ (100 µM). A positive control (PC) sample (mixture of phosphorylated and dephosphorylated α-casein) was run on the gel at the same time for confirmation that the gel is correctly working to separate the proteins with different phosphorylation states. The PC sample-loaded gel was stained with Coomassie Brilliant Blue.
